# Sex hormone mediated change on flexion reflex

**DOI:** 10.3389/fnins.2023.1263756

**Published:** 2023-12-21

**Authors:** Subaryani D. H. Soedirdjo, Yu-Chen Chung, Yasin Y. Dhaher

**Affiliations:** ^1^Department of Physical Medicine and Rehabilitation, University of Texas Southwestern Medical Center, Dallas, TX, United States; ^2^Department of Biomedical Engineering, University of Texas Southwestern Medical Center, Dallas, TX, United States; ^3^Peter O’Donnell Jr. Brain Institute, University of Texas Southwestern Medical Center, Dallas, TX, United States

**Keywords:** flexion reflex, menstrual cycle, estradiol, progesterone, innocuous

## Abstract

It has been shown that estrogen and progesterone receptors are expressed in the spinal cord; therefore, fluctuation in their concentrations may affect the spinal network and modulate the control of movement. Herein, we assessed the neuro-modulatory effect of sex hormones on the polysynaptic spinal network by using a flexion reflex network as a model system. Twenty-four healthy eumenorrheic women (age 21–37 years) were tested every other day for one menstrual cycle. Serum estradiol and progesterone were acquired at the time of testing. The flexion reflex of the tibialis anterior was elicited by sending an innocuous electrical stimulus directly to the posterior tibial nerve or plantar cutaneous afferent. Analyses were performed for each menstrual cycle phase: the follicular phase and the luteal phase. Increases in estradiol or progesterone concentrations were not associated with reflex duration or root mean squared (RMS) amplitude in either the follicular or luteal phases. In the luteal phase, an increase in the estradiol concentration was associated with a longer latency of the reflex (*b* = 0.23, *p* = 0.038). The estradiol × progesterone interaction was found towards significance (*b* = −0.017, *p* = 0.081). These results highlight the potential synergistic effect of estradiol and progesterone and may provide indirect confirmatory evidence of the observed modulatory effect.

## 1 Introduction

The actions of sex hormones on neuronal excitability at the spinal level remain elusive. For example, the presence of estrogen receptors in the lumbosacral spinal cord (Papka and Mowa, 2003), the ventral and dorsal horns (Dun et al., 2009), combined with evidence of estradiol’s role in synaptic transmission in cortical circuits (Srivastava, 2012; Sellers et al., 2015) suggests that estradiol may influence the neuronal excitability of spinal circuits. It has been reported that the cellular effect of sex hormone estradiol is expressed from minutes (non-genomic effects) to days (genomic effects) (Colciago et al., 2020). The non-genomic effect, for example, acts on modulating gamma aminobutyric acid (GABA), N-methyl-D-aspartate receptors, and glutamate receptors (Foy et al., 1999; Smith and Woolley, 2004; Micevych and Mermelstein, 2008), which are the same neurobiological constituencies that regulate spinal motoneurons. On the other hand, progesterone can influence neural systems through the GABAergic effect of a progesterone metabolite, at least in cortical neurons (Smith et al., 2002). Although several studies examined sex hormones-mediated neuronal excitability in animals (Warren et al., 1995; Foy et al., 1999; Parducz et al., 2006), explorations of similar effects in humans are limited. Data from our group indicated that, at rest, spinal motoneurons excitability assessed using the H-reflex paradigm (monosynaptic pathway) did not show a significant dependence on acute changes (within a cycle) of sex hormone levels (estradiol and progesterone) in female participants (Casey et al., 2016). It is not, however, known if the observed lack of effect at the motor neuron level will also be expressed at the circuit level. Indeed, during activation, motor neurons receive inputs from multiple sources: descending motor commands, peripheral sensory signals, and inter-neuronal inputs (Bourane et al., 2015). The lack of evidence that sex hormones affect motor neurons does not preclude the potential that estradiol and progesterone have an effect at the circuit level.

In this study, we sought to explore the neuro-modulatory effect of sex hormones (estradiol and progesterone) on a spinal circuit by testing their effects on a known polysynaptic spinal network, the flexion reflex network, as a model system (Sandrini et al., 2005). This type of reflex is composed of two excitatory components separated by a silent period (Meinck et al., 1981). The first component (RII) is mediated by the activation of low threshold mechano-afferents (LTMR); through Aβ fibers. The second component (RIII) is a longer latency response and is mediated by Aδ fiber afferents involved in pain perception (Hugon, 1973; Ertekin et al., 1975). Indeed, several studies have reported changes in the perception of pain across the menstrual cycle (Tassorelli et al., 2002; Bartley and Rhudy, 2013). The RII component, on the other hand, is involved in a sensory feedback circuit that plays an important role in the control of motor neuron activation (Böhm and Wyart, 2016); Aβ pathways are involved in the regulation of the step cycle phase in human walking (van Wezel et al., 2000). An examination targeting this class of reflexes will reveal the potential functional implications of the fluctuating sex hormones expressed at the spinal motor circuit level.

The two sex hormones that fluctuate across the menstrual cycle are estradiol and progesterone. During the follicular phase, the estradiol concentration increases from its minimum at menses to its maximum at peri-ovulation. Following a decrease at ovulation, the estradiol concentration fluctuates during the luteal phase along with an increase in progesterone concentration (Reed and Carr, 2000). This temporal profile makes the assessment of the effect of estradiol on flexion reflex response in humans straightforward: the examination is conducted in the first half of the menstrual cycle, a state with low progesterone concentrations. The exploration of the effect of progesterone on flexion reflex circuit is more complex. The increase in progesterone in the second half of the menstrual cycle is expressed in the presence of background levels of estradiol. Thus, we chose an experimental design to examine the effect of sex hormones on flexion reflex response by acquiring data every other day in the cycle.

The goal of our study was to evaluate the interplay between the flexion reflex response and the fluctuation of sex hormones across the menstrual cycle. An innocuous stimulation of the posterior tibial nerve or the sole of the foot was applied to activate the flexion reflex arc (Michel et al., 2008; Gervasio et al., 2018) either through the direct stimulation of sensory fibers in the nerve and the low threshold mechanoreceptors (LTMR) driving the nerve signal, respectively. Based on the reported data on cortical neurons, we hypothesized that an acute increase in estradiol during the follicular phase would augment the flexion reflex response. In addition, we hypothesized that an increase in progesterone during the luteal phase would counteract the effect of estradiol.

## 2 Methods

Healthy young eumenorrheic women from similar demographic groups were recruited for this study. Every other day testing sessions were conducted, and serum samples were acquired. Two innocuous peripheral nerve stimulation paradigms were employed to activate the sensory fibers, and the flexion reflex response was recorded. No sample size analysis was performed prior to the testing due to lack of data on the link between sex hormons and polysynaptic reflexes.

### 2.1 Participants

Northwestern University and the University of Texas Southwestern Medical Center Institutional Review Boards approved the protocol. Fifty-seven healthy eumenorrheic females with moderate physical activity and no history of lower limb disorder were enrolled in the study. Women with moderate physical activity were defined as those who exercised less than 7 hours per week and were not participating in competitive-level sports. We had to exclude a total of 33 women: two due to lack of adherence to the protocol, two due to sensitivity to electrical stimulation, four due to the absence of estradiol and/or progesterone fluctuations across the menstrual cycle, 12 withdrew from the study, and 13 decided not to participate in any part of the study after being enrolled. Thus, the results presented here are based on data acquired from 24 healthy eumenorrheic women (age: 27.0 ± 4.4 years; BMI: 24.8 ± 4.8 kg/m^2^; cycle length: 30.3 ± 3.8 days; means ± SD) involved involved in the study. All participants signed the written informed consent form and were free to participate.

Women who exercised more than 7 h per week or participated in competitive level sports were excluded because of the high rate of undiagnosed menstrual dysfunction in this population (Gibbs et al., 2013). Participants were asked to maintain stable exposure to caffeine, alcohol, and exercise during the 12 hours before each testing session to minimize changes in reflex excitability (Motl and Dishman, 2003; Walton et al., 2003; von Dincklage et al., 2007).

### 2.2 Experimental protocol and procedures

Each participant was tested every other day for one menstrual cycle (about 15 to 20 testing sessions depending on the length of the cycle). The personnel performing the testing were blinded to subject’s menstrual cycle phase. The start date of the testing was assigned randomly. The tesing time was scheduled based on participant’s preference and maintained at the same time of the day throughout the testing sessions to minimize the effect of diurnal fluctuations of hormone levels (Bao et al., 2003; Bungum et al., 2011) and circadian variations of the flexion reflex (Sandrini et al., 1986a).

### 2.3 Hormonal levels

The hormone concentrations were obtained from blood samples collected by means of venipuncture. The samples were collected at each testing session from antecubital area of the participant’s arm using a 5 mL Vacutainer serum separator tube (BD, Franklin Lakes, New Jersey). Once the tube was filled, the sample was mixed well and allowed to clot. The samples obtained at Northwestern were processed at Northwestern Memorial Hospital Outpatient Laboratory and the samples obtained at UT Southwestern were sent to MedFusion. The hormonal concentrations analyzed were estradiol and progesterone.

### 2.4 Testing condition and electrodes placement

The participants were placed in a supine position on a padded examination table. Each participant was instructed to choose a comfortable position for her head and arms. The position was recorded so it could be replicated at subsequent testing sessions. They were instructed not to activate their muscle prior, during, and after the stimulation paradigm.

Bipolar surface electromyogram (sEMG) signals were detected from the tibialis anterior either with pre-amplified electrodes (DE-2.1 Bagnoli single differential, interelectrode distance: 10 mm, contact dimensions 10 × 1 mm) or bipolar Ag/AgCl pre-gelled electrodes (diameter 10 mm, inter electrode distance 20 mm). The electrode type for each subject was consistent throughout the testing sessions.

Prior to electrode placement, the skin was cleaned with alcohol, slightly abraded (Nuprep®, Weaver and Company, CO, United States), and cleaned with water to remove any residue. The locations of the electrodes were recorded and marked with a waterproof or blacklight marker to ensure the same electrode location in each session. The signals were digitized with a Micro1401 Data Acquisition Unit (Cambridge Electronic Design, United Kingdom) at a rate of 2,000 Hz.

### 2.5 Elicitation of the flexion reflex

Classically, examination of the flexion reflex pathway in humans used two unique paradigms: peripheral nerve electrical stimulation and electrical stimulation to the skin in the sole of the foot (Van Wezel et al., 1997; Knikou, 2007). In this study we incorporated both paradigms, in almost evenly split cohorts, 11 and 13 subjects respectively. Implementation of both paradigms allows for a tertiary analysis to compare the consistency in the flexion reflex response across the two paradigms.

Posterior tibial nerve stimulation: The flexion reflex of 11 participants was elicited by delivering a train of eight pulses (2 ms pulse width, 200 Hz) using a Grass Stimulator S48 (Grass Instruments, Quincy, MA) connected to an isolation stimulus unit (SIU5, Grass Instruments) and passed through constant current unit (CCU1, Grass Instruments). The bipolar stimulating electrode (electrode diameter 0.8 cm, inter electrode distance 2 cm) was placed over the posterior tibial nerve located posterior and inferior to the medial malleolus, with the anode positioned posteriorly. Optimal placement of the stimulating electrode was found by moving the stimulating electrode over the nerve until a low-current stimulus elicited the reflex response. To find the reflex threshold, the stimulus intensity was increased manually with 0.2 mA increments until a distinguishable and stable response was clearly seen. Then a maximum of 12 stimuli with an intensity at the reflex threshold were delivered to the subjects with a 30–35 s random inter-stimulus interval until at least four responses were obtained.

Plantar cutaneous afferents stimulation: For the remaining 13 participants, a pair of square gel electrodes (5 cm x 5 cm) were used. The cathode was placed over the area between the first and second metatarsal, while the anode was placed on the arch of the foot. A train of five pulses (1 ms pulse width, 200 Hz) was delivered using a DS7A constant current stimulator (Digitimer Ltd., Hertfordshire, England). The reflex threshold was defined as the smallest intensity that elicited the reflex response. Then a maximum of 20 stimuli with an intensity 10% higher than the reflex threshold was delivered to the participant with a 30–35 s random inter-stimulus interval.

Post-hoc analysis indicated that within subjects, stimulation intensity across visits showed no statistically significant correlation on visits suggesting that there was no effect of habituation nor hormone concentration dependency.

### 2.6 Data analysis

**Figure 1:**
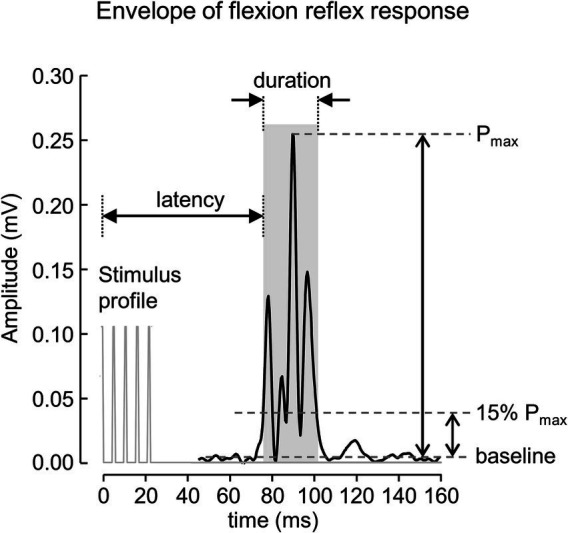
A rectified EMG envelope of muscle response obtained from one subject. The flexion reflex response is marked with gray area. The onset and offset of the response were defined as start and end of EMG envelope where the amplitude was greater than 15% of the peak amplitude measured from the baseline value.

The signals were processed offline using Matlab software (R2019a. The MathWorks Inc., MA, United States). A 4th order zero phase Butterworth bandpass filter (final cut off frequency 20–500 Hz) was applied to the signal to attenuate movement artifacts and other interference. The envelopes of rectified EMG signals extracted using a 4th order zero phase Butterworth low-pass filter with a final cut off frequency at 200 Hz were used to define the onset and offset of the reflex response. The start and end of the reflex response were determined when the envelope of the reflex signal crossed 15% of the peak amplitude measured from the baseline value (Figure 1). The baseline value was defined as the average of the 250 ms EMG envelopes before the onset of the stimulus. The onset and offset of the reflex response were used to calculate root mean squared (RMS) value, duration, and latency, measured from the onset of the stimulus. The average value of each parameter for all identified reflex responses was then calculated and used for further analysis.

The magnitude of the flexion reflex reflex (RMS value), duration, and latency were analyzed at: (1) the follicular phase, where the estradiol concentration increased from low level to its maximum while the progesterone concentrations remained low, and (2) the luteal phase, where the progesterone increased from low level to its maximum along with background estradiol concentration.

### 2.7 Statistical analyses

Data are presented as mean ± SD. The statistical analysis for this study was performed in the R language (version 4.2.2, R Foundation for Statistical Computing, Vienna, Austria). To evaluate the effect of changes in hormones level on reflex RMS, duration, and latency, a generalized estimating equation (GEE) with log link function and exchangeable correlation structure was employed using geepack R package (Ballinger, 2004; Halekoh et al., 2006). Each of the flexion reflex parameters was simultaneously regressed on estradiol, progesterone, and the estradiol × progesterone interaction. The estradiol concentration in ng/ml was used in GEE to keep the units consistent between estradiol and progesterone. Significance was accepted for *p* values <0.05.

## 3 Results

### 3.1 Hormone profile

**Figure 2:**
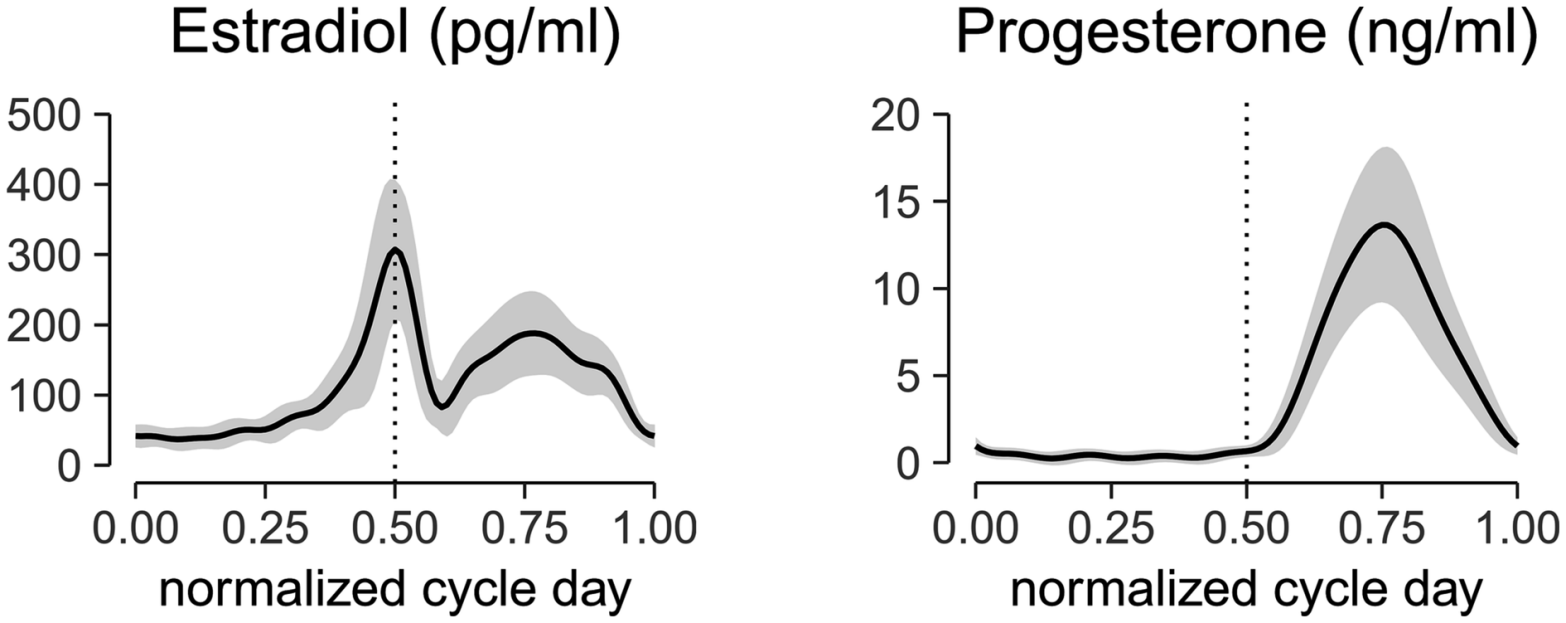
Aligned hormonal profiles from ovulatory cycles of 24 subjects tested in the study. Serum measurements were collected every other day for 1 cycle. Cycle day was normalized to the cycle length and centered at the peak estradiol concentration. Normalized centered cycle day = 0 corresponds to the first day of menses, 0.5 corresponds to the peak of estradiol concentrations, 0.75 corresponds to the peak of progesterone concentrations, and 1 corresponds to the last day of the cycle. The solid line represents the mean of all subjects, and the ribbon represents the standard deviation. The mid-cycle is marked with a dashed vertical line.

The estradiol and progesterone profiles obtained from a non-linear mixed effect model with harmonic terms (Albert and Hunsberger, 2005) is shown in Figure 2. The cycle day was normalized to the cycle length and centered such that 0 corresponds to first day of menses, 0.5 corresponds to peak estradiol, 0.75 corresponds to peak progesterone, and 1.0 corresponds to last day of the cycle. Estradiol concentrations at the first day of menses, peri-ovulatory (peak estradiol concentration), and mid-luteal (peak progesterone concentration) time points were 41.7 ± 16.2 pg/mL, 307.23 ± 99.2 pg/mL, and 186.7 ± 60.2 pg/mL, respectively. The corresponding progesterone concentrations at those time points were 1.0 ± 0.5 ng/mL, 0.7 ± 0.3 ng/mL, and 13.6 ± 4.4 ng/mL. These concentrations are consistent within the range of estradiol and progesterone levels reported in the literature (Sehested, 2000; Marsh et al., 2011; Mumford et al., 2012).

### 3.2 Fexion reflex response

**Figure 3:**
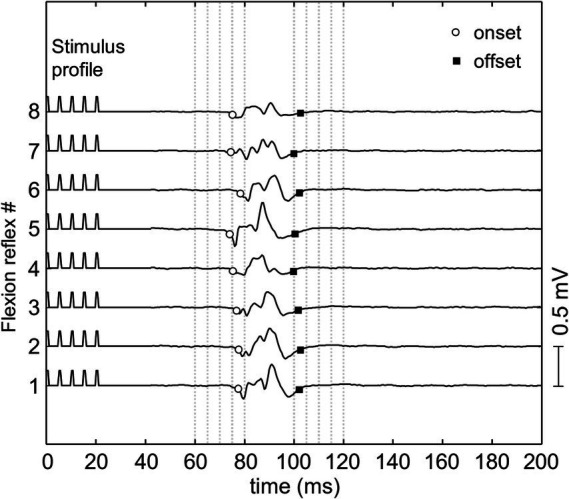
The flexion reflex response on tibialis anterior muscle obtained from one subject. The reflex was elicited using a train of five pulses at 200 Hz and pulse width of 1 ms delivered to the sole of the foot with inter-stimulus interval of 30–35 s.

A typical flexion reflex response is shown in Figure 3. The average stimulation intensity used in this study was 7.2 ± 3.5 mA. The stimulation intensity applied on the posterior tibial nerve stimulation ranged from 2.5 to 15.0 mA and the stimulation intensity applied on the plantar cutaneous afferents ranged from 3.0 to 20.9 mA across participants. Participants reported no pain during and following stimulation. Post-hoc analysis on the stimulation intensity indicated no significant difference between the two groups (*p* > 0.05). The average response rate of posterior tibial nerve stimulation and plantar cutaneous afferents stimulation were 78% (8 responses out of 10 stimuli) and 47% (9 responses out of 20 stimuli) respectively. A non-parametric Kurskal-Wallis test showed that reflex latency and duration at menses did not differ between the two stimulation paradigms: posterior tibial nerve and plantar cutaneous afferents stimulation (Table 1, *p* > 0.05), suggesting that both paradigms activated an equivalent flexion reflex pathway.

**Table 1:** Latency and duration of flexion reflex elicited using posterior tibial nerve stimulation and plantar cutaneous afferents stimulation at menses.

Variables	Stimulation paradigm		*p* value
	Posterior tibial nerve	Plantar cutaneous afferents	
Latency (ms)	79.65 ± 7.24	84.51 ± 4.19	0.193
Duration (ms)	33.43 ± 9.83	41.45 ± 15.07	0.305

### 3.3 Sex hormone fluctuation and flexion reflex

A GEE model on data collected during the follicular phase showed that estradiol concentration was not associated with reflex latency, duration, and RMS magnitude (Table 2). In the luteal phase, reflex duration and RMS magnitude were not associated with fluctuation of either estradiol or progesterone. As indicated in Table 2, in the luteal phase, the GEE revealed a main effect of estradiol (*b* = 0.228, *p* = 0.038) on reflex latency. Our data suggests that increases in estradiol in the luteal phase predicts longer reflex latency better than chance. Increases in progesterone make this association slightly less pronounced (*b* = −0.017, *p* = 0.081).

**Table 2:** Generalized estimating equation of the main effect estradiol, progesterone, and estradiol × progesterone interaction on the latency, duration, and RMS of reflex response across the menstrual cycle.

Variables	Generalized Estimating Equation – *b* (*p* value)		
	Estradiol (ng/ml)	Progesterone (ng/ml)	Interaction
Follicular phase			
Latency (ms)	0.036 (0.240)	–	–
Duration (ms)	0.072 (0.510)	–	–
RMS (mV)	−0.060 (0.870)	–	–
Luteal phase			
Latency (ms)	0.228 (0.038)*	0.001 (0.532)	−0.017 (0.081)
Duration (ms)	0.535 (0.140)	0.007 (0.160)	−0.028 (0.300)
RMS (mV)	−0.885 (0.510)	0.018 (0.320)	−0.045 (0.640)

The data was analyzed within the follicular phase (estradiol concentrations increase to its maximum while the progesterone concentrations were low) and the luteal phase (progesterone concentration increased concomitantly with estradiol).

## 4 Discussion

This study set out with the aim of assessing the effect of fluctuating sex hormones on polysynaptic spinal circuits during quiescent state represented by the well-studied flexion reflex network in humans. Two paradigms employed in this study used stimulation profiles that selectively stimulate sensory fibers (Veale et al., 1973) and target the polysynaptic spinal network mediated by sensory fibers (Shahani, 1970). Our primary hypothesis was that the increase in estradiol concentration during the first half of the menstrual cycle would increase the magnitude of flexion reflex response and the increase in progesterone concentration during the second half would inhibit the effect of estradiol. Our results showed that the duration and RMS amplitude of the flexion reflex response were not modulated across the menstrual cycle. Contrary to our hypothesis, increases in estradiol concentrations from menses to the peri-ovulatory phase were not associated with the RMS amplitude of the flexion reflex. Interestingly, in the luteal phase, increases in the estradiol concentrations were associated with increases in the latency of flexion reflex.

### 4.1 Sex hormones and the flexion reflex pathway

The latencies of the reflex responses observed in this study were longer than 60 ms, the natural separation time between the RII and RIII responses (Andersen, 2007). Hence, the observed reflex response can be categorized as the RIII response mediated by Aδ fibers, although the stimulus intensities delivered to the participants were below the pain threshold. The Aδ fiber afferents mainly terminate in laminae I and II, where estrogen receptors (ERα, ERβ, and G protein-coupled receptor 30) are present (Shughrue et al., 1997; VanderHorst et al., 2005; Takanami et al., 2010). In an animal model, estrogen was shown to modulate nociceptive transmission (Li et al., 2009). Specifically, ERα and ERβ were shown to inhibit and enhance nociceptive transmission, respectively (Coulombe et al., 2011). The invariant flexion reflex response during the follicular phase observed in this study may likely be due to the activation of both estrogen receptors, resulting in zero total effect of estradiol.

In the luteal phase, we observed a significant association between estradiol concentration and the latency of the reflex. Although the magnitude of the association is modest, it is tempting to speculate that with the increase in estradiol concentrations in the luteal phase the processing of flexion reflex may be involved to a more complex network when compared to the neuronal circuit at the follicular phase, prior to projection into the motor neuron (Abraira and Ginty, 2013). The mechanism of estradiol’s effect on the latency of the flexion reflex in the presence of background progesterone warrants further examination.

Given the lack of association between estradiol and the latency of the flexion reflex during the follicular phase, one could argue that the observed association between estradiol and latency during the luteal phase is likely facilitated by the presence of progesterone. Histological evidence from animal models indicates that progesterone receptors are present in the lamina IX of the spinal cord (Labombarda et al., 2000), where motor neurons are located (Osseward and Pfaff, 2019), likely modulating changes in the flexion reflex arc. The isolated neuronal effect of progesterone has been explored, for the most part, in cortical structures, specifically hippocampal neuron (Woolley and McEwen, 1993; Weiland and Orchinik, 1995; Foy et al., 2010), with a primary effect in modulating the synaptic plasticity and regulation of GABA_A_ receptors. In the spinal cord, results from the rodent models were restricted to the neuroinflammatory effect of progesterone and its role in neurodegeneration after trauma (Thomas et al., 1999; Gonzalez Deniselle et al., 2002). Specifically, progesterone has been shown to be neuroprotective through brain derived neurotrophic factor (BDNF) mRNA upregulation (González et al., 2004; De Nicola et al., 2006), downregulation of neuronal Na, K-ATPase regulatory subunits (Labombarda et al., 2002), and promotion of myelin formation (Labombarda et al., 2010). In a spinal cord injury animal model, progesterone was shown to prevent upregulation of N-methyl-D-aspartate receptor subunits, an important component that induces pain produced by tactile stimuli (Coronel et al., 2011). Allopregnanolone, a progesterone metabolite, is also shown to increase neuroinhibitory effect by upregulation of GABA_A_ receptors (Twyman and Macdonald, 1992). It is unclear, however, what the underlying mechanism is for the interaction of progesterone with the function of estradiol at the level of the neural circuit.

There have been limited examinations on the synergistic effects of progesterone and estradiol on neuronal properties. Co-treatment of estradiol and progesterone was shown to reduce the BDNF levels in the hippocampus, while treatment with estradiol alone did not affect the levels of BDNF (Gibbs, 1999). Co-treatment of estradiol and progesterone was also shown to significantly enhance voltage-gated calcium conductance in hippocampal pyramidal neurons (Joëls and Karst, 1995). However, examinations in animal models on the probable estradiol-progesterone combined facilitatory effect on spinal neuronal networks are nonexistent. Correlative evidence seems to indicate that administration of progesterone on estrogen-primed animals is shown to facilitate lordosis (Whalen, 1974; Kow et al., 1979), a reflex response triggered by a touch stimulus (Kow and Pfaff, 1976), possibly through upregulation of estrogen-inducible progesterone receptors (Monks et al., 2001). Our *in vivo* data indicates that estradiol, with the presence of progesterone, may have a neurophysiologic effect at the spinal level, manifested by the finding that an increase in estradiol concentration in the luteal phase was associated with an increase of reflex latency. While the increase in estradiol concentration during the follicular phase was not associated with any reflex properties, a significant effect emerged during the luteal phase, as well as a trend toward significance for estradiol × progesterone effect. This finding may suggest the important role of progesterone in enhancing the effect of estradiol. This potential interaction warrants further examination.

### 4.2 Clinical implication

It has been suggested the interneuron circuits involved in the flexion reflex are partly shared with the interneurons responsible for the central pattern generator for locomotion (Windhorst, 2021). Specifically, the afferents in the glabrous skin of the foot have an important role in sensing the foot’s contact with the ground and may contribute to standing balance and movement control (Kennedy and Inglis, 2002). In disease states such as in spinal cord injury and traumatic brain injury, administration of estradiol and progesterone has been proposed as a potential therapeutic option (Wright et al., 2007; Shvetcov et al., 2023) due to their important role as neuroprotective agent and the reported recovery of locomotor function in animal models (Thomas et al., 1999; Sribnick et al., 2003).

Our findings on the effect of estradiol on the flexion reflex latency would likely have functional significance during states where estradiol concentration is higher than those observed during the natural menstrual cycle. Recall that our regression coefficient between estradiol (ng/ml) and reflex latency (ms) was estimated to be 0.23, leading to a functionally insignificant within-cycle change in latency of 0.092 ms due to estradiol (a maximum change of 0.4 ng/mL). However, these effects may be significant in the states where higher estradiol levels are expected. During pregnancy, for example, the estradiol level has been reported to reach 30 ng/mL (Schock et al., 2016), leading to a probable change of a latency up to 7 ms during this state. The functional significance in the latency change of this magnitude is still under debate. However, in the context of a pathological state, it has been shown that individuals with chronic ankle instability expressed a 7 ms difference in TA latency between the affected and unaffected sides in response to perturbation (Sousa et al., 2019). Hence, our results may provide a basis for future examinations in physiological states in which the estradiol concentration is significantly higher than the average change observed during the menstrual cycle.

### 4.3 Methodological consideration

While the findings in this study contribute in several ways to our understanding of the effect of sex hormones on polysynaptic reflex networks, these results are limited to healthy pre-menopausal women and may need to be examined in females with neurological injuries (for example, spinal cord injury). In addition, the complex interactions between sex hormones, and other reproduction-associated hormones (luteinizing hormone, follicle-stimulating hormone, relaxin), and neurotransmitters, such as glutamate, serotonin, and dopamine (Barth et al., 2015) were not considered in this study. Both animal and human studies have shown that the interneurons involved in the nociceptive component of the flexion reflex (RIII response) are modulated by dopaminergic, cholinergic and GABAergic neural transmission (Mondrup and Pedersen, 1984; Sandrini et al., 1986b; Clemens and Hochman, 2004; Gerdelat-Mas et al., 2007). It is likely that during the luteal phase, the observed estradiol-mediated latency changes of flexion reflex may be attributed to the changes in neurotransmitters. In addition, and despite of the reported conflicting findings, a state of anxiety may modulate the nociceptive component of flexion reflex (RIII response) (French et al., 2005). In this study, multiple ways were implemented to minimize participants’ anxiety during testing. Participants were instructed to choose a comfortable position for their head and arms, fully relax and remain awake, and their muscle activation levels were monitored by the investigators. In addition, the testing was conducted in a quiet examination room with dim lighting. However, future examination incorporating measures of anxiety, such a questionnaire, and heart rate or skin conduction rate monitoring, may be required.

To our knowledge, this is the first study to investigate the neuromodulatory effect of sex hormones specifically on the polysynaptic spinal network activated by innocuous stimulation of sensory fibers on the sole of the foot and the posterior tibial nerve. Together with supraspinal commands, external inputs such as LTMR, and internal inputs from muscle spindles and the Golgi tendon organ, the sensation of pain constructs a sensory feedback circuit in the spinal cord and plays important roles in the control of motor neuron activation (Böhm and Wyart, 2016). Acute modulation of the sex hormones may alter the polysynaptic spinal circuit and may have implication for the restoration of motor function.

## Data availability statement

The raw data supporting the conclusions of this article will be made available by the authors, without undue reservation.

## Author contributions

SS: Data curation, Visualization, Writing – original draft, Writing – review & editing, Investigation. Y-CC: Data curation, Visualization, Writing – original draft, Writing – review & editing, Investigation. YD: Conceptualization, Funding acquisition, Methodology, Resources, Supervision, Writing – review & editing, Writing – original draft.

## References

[ref1] Abraira V. E. Ginty D. D. (2013). The sensory neurons of touch. Neuron 79, 618–639. doi: 10.1016/j.neuron.2013.07.051, PMID: 23972592 PMC3811145

[ref2] Albert P. S. Hunsberger S. (2005). On analyzing circadian rhythms data using nonlinear mixed models with harmonic terms. Biometrics 61, 1115–1120. doi: 10.1111/j.0006-341X.2005.464_1.x, PMID: 16401286

[ref3] Andersen O. K. (2007). Studies of the organization of the human nociceptive withdrawal reflex: focus on sensory convergence and stimulation site dependency. Acta Physiol. 189, 1–35. doi: 10.1111/j.1748-1716.2007.01706.x, PMID: 17439638

[ref4] Ballinger G. A. (2004). Using generalized estimating equations for longitudinal data analysis. Organ. Res. Methods 7, 127–150. doi: 10.1177/1094428104263672

[ref5] Bao A.-M. Liu R.-Y. van Someren E. J. W. Hofman M. A. Cao Y.-X. Zhou J.-N. (2003). Diurnal rhythm of free estradiol during the menstrual cycle. Eur. J. Endocrinol. 148, 227–232. doi: 10.1530/eje.0.1480227, PMID: 12590642

[ref6] Barth C. Villringer A. Sacher J. (2015). Sex hormones affect neurotransmitters and shape the adult female brain during hormonal transition periods. Front. Neurosci. 9:37. doi: 10.3389/fnins.2015.00037, PMID: 25750611 PMC4335177

[ref7] Bartley E. J. Rhudy J. L. (2013). Comparing pain sensitivity and the nociceptive flexion reflex threshold across the mid-follicular and late-luteal menstrual phases in healthy women. Clin. J. Pain 29, 154–161. doi: 10.1097/AJP.0b013e31824c5edb, PMID: 22688607

[ref8] Böhm U. L. Wyart C. (2016). Spinal sensory circuits in motion. Curr. Opin. Neurobiol. 41, 38–43. doi: 10.1016/j.conb.2016.07.007 27573214

[ref9] Bourane S. Grossmann K. S. Britz O. Dalet A. Del Barrio M. G. Stam F. J.. (2015). Identification of a spinal circuit for light touch and fine motor control. Cells 160, 503–515. doi: 10.1016/j.cell.2015.01.011, PMID: 25635458 PMC4431637

[ref10] Bungum L. Jacobsson A. K. Rosn F. Becker C. Yding Andersen C. Gner N.. (2011). Circadian variation in concentration of anti-Mllerian hormone in regularly menstruating females: relation to age, gonadotrophin and sex steroid levels. Hum. Reprod. 26, 678–684. doi: 10.1093/humrep/deq380, PMID: 21227943

[ref11] Casey E. Reese M. Okafor E. Chun D. Gagnon C. Nigl F.. (2016). Influence of menstrual cycle and Oral contraceptive phase on spinal excitability. PM&R 8, 860–868. doi: 10.1016/j.pmrj.2016.01.013, PMID: 26872589 PMC5278436

[ref12] Clemens S. Hochman S. (2004). Conversion of the modulatory actions of dopamine on spinal reflexes from depression to facilitation in D3 receptor knock-out mice. J. Neurosci. 24, 11337–11345. doi: 10.1523/JNEUROSCI.3698-04.2004, PMID: 15601940 PMC2731231

[ref13] Colciago A. Bonalume V. Melfi V. Magnaghi V. (2020). Genomic and non-genomic action of Neurosteroids in the peripheral nervous system. Front. Neurosci. 14. doi: 10.3389/fnins.2020.00796 PMC7403499 32848567

[ref14] Coronel M. F. Labombarda F. Villar M. J. De Nicola A. F. González S. L. (2011). Progesterone prevents allodynia after experimental spinal cord injury. J. Pain 12, 71–83. doi: 10.1016/j.jpain.2010.04.013, PMID: 20675200

[ref15] Coulombe M. A. Spooner M. F. Gaumond I. Carrier J. C. Marchand S. (2011). Estrogen receptors beta and alpha have specific pro- and anti-nociceptive actions. Neuroscience 184, 172–182. doi: 10.1016/j.neuroscience.2011.02.057, PMID: 21377511

[ref16] De Nicola A. F. Gonzalez S. L. Labombarda F. Deniselle M. C. G. Garay L. Guennoun R.. (2006). Progesterone treatment of spinal cord injury: effects on receptors, neurotrophins, and myelination. J. Mol. Neurosci. 28, 3–16. doi: 10.1385/JMN:28:1:3, PMID: 16632872

[ref17] Dun S. L. Brailoiu G. C. Gao X. Brailoiu E. Arterburn J. B. Prossnitz E. R.. (2009). Expression of estrogen receptor GPR30 in the rat spinal cord and in autonomic and sensory ganglia. J. Neurosci. Res. 87, 1610–1619. doi: 10.1002/jnr.21980, PMID: 19125412 PMC2692324

[ref18] Ertekin C. Ertekin N. Karcioglu M. (1975). Conduction velocity along human nociceptive reflex afferent nerve fibres. J. Neurol. Neurosurg. Psychiatry 38, 959–965. doi: 10.1136/jnnp.38.10.959, PMID: 1202167 PMC492130

[ref19] Foy M. R. Baudry M. Akopian G. K. Thompson R. F. (2010). Regulation of hippocampal synaptic plasticity by estrogen and progesterone. Vitam. Horm., 219–239. doi: 10.1016/S0083-6729(10)82012-6, PMID: 20472141

[ref20] Foy M. R. Xu J. Xie X. Brinton R. D. Thompson R. F. Berger T. W. (1999). 17β-estradiol enhances NMDA receptor-mediated EPSPs and long-term potentiation. J. Neurophysiol. 81, 925–929. doi: 10.1152/jn.1999.81.2.925, PMID: 10036289

[ref21] French D. J. France C. R. France J. L. Arnott L. F. (2005). The influence of acute anxiety on assessment of nociceptive flexion reflex thresholds in healthy young adults. Pain 114, 358–363. doi: 10.1016/j.pain.2004.12.034, PMID: 15777861

[ref22] Gerdelat-Mas A. Simonetta-Moreau M. Thalamas C. Ory-Magne F. Slaoui T. Rascol O.. (2007). Levodopa raises objective pain threshold in Parkinson’s disease: a RIII reflex study. J. Neurol. Neurosurg. Psychiatry 78, 1140–1142. doi: 10.1136/jnnp.2007.120212, PMID: 17504881 PMC2117570

[ref23] Gervasio S. Laursen C. B. Andersen O. K. Hennings K. Spaich E. G. (2018). A novel stimulation paradigm to limit the habituation of the nociceptive withdrawal reflex. IEEE Trans. Neural Syst. Rehabil. Eng. 26, 1100–1107. doi: 10.1109/TNSRE.2018.2828221, PMID: 29752246

[ref24] Gibbs R. B. (1999). Treatment with estrogen and progesterone affects relative levels of brain-derived neurotrophic factor mRNA and protein in different regions of the adult rat brain. Brain Res. 844, 20–27. doi: 10.1016/S0006-8993(99)01880-6, PMID: 10536257

[ref25] Gibbs J. C. Williams N. I. De Souza M. J. (2013). Prevalence of individual and combined components of the female athlete triad. Med. Sci. Sport. Exerc. 45, 985–996. doi: 10.1249/MSS.0b013e31827e1bdc, PMID: 23247706

[ref26] Gonzalez Deniselle M. C. Lopez Costa J. J. Gonzalez S. L. Labombarda F. Garay L. Guennoun R.. (2002). Basis of progesterone protection in spinal cord neurodegeneration. J. Steroid Biochem. Mol. Biol. 83, 199–209. doi: 10.1016/S0960-0760(02)00262-5, PMID: 12650717

[ref27] González S. L. Labombarda F. González Deniselle M. C. Guennoun R. Schumacher M. De Nicola A. F. (2004). Progesterone up-regulates neuronal brain-derived neurotrophic factor expression in the injured spinal cord. Neuroscience 125, 605–614. doi: 10.1016/j.neuroscience.2004.02.024, PMID: 15099674

[ref28] Halekoh U. Højsgaard S. Yan J. (2006). The R package geepack for generalized estimating equations. J. Stat. Softw. 15, 1–11. doi: 10.18637/jss.v015.i02

[ref29] Hugon M. (1973). Exteroceptive Reflexes to Stimulation of the Sural Nerve in Normal Man. In Human Reflexes, Pathophysiology of Motor Systems, Methodology of Human Reflexes. eds. Karger AG S. (pp. 713–729). doi: 10.1159/000394186

[ref30] Joëls M. Karst H. (1995). Effects of estradiol and progesterone on voltage-gated calcium and potassium conductances in rat CA1 hippocampal neurons. J. Neurosci. 15, 4289–4297. doi: 10.1523/jneurosci.15-06-04289.1995, PMID: 7790911 PMC6577704

[ref31] Kennedy P. M. Inglis J. T. (2002). Distribution and behaviour of glabrous cutaneous receptors in the human foot sole. J. Physiol. 538, 995–1002. doi: 10.1113/jphysiol.2001.013087, PMID: 11826182 PMC2290100

[ref32] Knikou M. (2007). Plantar cutaneous input modulates differently spinal reflexes in subjects with intact and injured spinal cord. Spinal Cord 45, 69–77. doi: 10.1038/sj.sc.3101917, PMID: 16534501 PMC1764031

[ref33] Kow L. M. Montgomery M. O. Pfaff D. W. (1979). Triggering of lordosis reflex in female rats with somatosensory stimulation: quantitative determination of stimulus parameters. J. Neurophysiol. 42, 195–202. doi: 10.1152/jn.1979.42.1.195, PMID: 430110

[ref34] Kow L.-M. Pfaff D. W. (1976). Sensory requirement for the lordosis reflex in female rats. Brain Res. 101, 47–66. doi: 10.1016/0006-8993(76)90987-2, PMID: 1244220

[ref35] Labombarda F. Gonzalez S. L. Gonzalez Deniselle M. C. Guennoun R. Schumacher M. De Nicola A. F. (2002). Cellular basis for progesterone neuroprotection in the injured spinal cord. J. Neurotrauma 19, 343–355. doi: 10.1089/089771502753594918 11939502

[ref36] Labombarda F. Guennoun R. Gonzalez S. Roig P. Lima A. Schumacher M.. (2000). Immunocytochemical evidence for a progesterone receptor in neurons and glial cells of the rat spinal cord. Neurosci. Lett. 288, 29–32. doi: 10.1016/S0304-3940(00)01191-5 10869808

[ref37] Labombarda F. Meffre D. Delespierre B. Krivokapic-Blondiaux S. Chastre A. Thomas P.. (2010). Membrane progesterone receptors localization in the mouse spinal cord. Neuroscience 166, 94–106. doi: 10.1016/j.neuroscience.2009.12.012, PMID: 20025939

[ref38] Li L. Fan X. Warner M. Xu X.-J. Gustafsson J.-Å. Wiesenfeld-Hallin Z. (2009). Ablation of estrogen receptor α or β eliminates sex differences in mechanical pain threshold in normal and inflamed mice. Pain 143, 37–40. doi: 10.1016/j.pain.2009.01.005 19285805

[ref39] Marsh E. E. Shaw N. D. Klingman K. M. Tiamfook-Morgan T. O. Yialamas M. A. Sluss P. M.. (2011). Estrogen levels are higher across the menstrual cycle in African-American women compared with Caucasian women. J. Clin. Endocrinol. Metab. 96, 3199–3206. doi: 10.1210/jc.2011-1314, PMID: 21849524 PMC3200247

[ref40] Meinck H.-M. Piesiur-Strehlow B. Koehler W. (1981). Some principles of flexor reflex generation in human leg muscles. Electroencephalogr. Clin. Neurophysiol. 52, 140–150. doi: 10.1016/0013-4694(81)90161-9, PMID: 6167423

[ref41] Micevych P. E. Mermelstein P. G. (2008). Membrane estrogen receptors acting through metabotropic glutamate receptors: an emerging mechanism of estrogen action in brain. Mol. Neurobiol. 38, 66–77. doi: 10.1007/s12035-008-8034-z, PMID: 18670908 PMC2663000

[ref42] Michel J. Van Hedel H. J. A. Dietz V. (2008). Obstacle stepping involves spinal anticipatory activity associated with quadrupedal limb coordination. Eur. J. Neurosci. 27, 1867–1875. doi: 10.1111/j.1460-9568.2008.06145.x, PMID: 18371084

[ref43] Mondrup K. Pedersen E. (1984). The effect of the GABA-agonist, progabide, on stretch and flexor reflexes and on voluntary power in spastic patients. Acta Neurol. Scand. 69, 191–199. doi: 10.1111/j.1600-0404.1984.tb07801.x, PMID: 6377801

[ref44] Monks D. A. Arciszewska G. Watson N. V. (2001). Estrogen-inducible progesterone receptors in the rat lumbar spinal cord: regulation by ovarian steroids and fluctuation across the estrous cycle. Horm. Behav. 40, 490–496. doi: 10.1006/hbeh.2001.1717 11716578

[ref45] Motl R. W. Dishman R. K. (2003). Acute leg-cycling exercise attenuates the h-reflex recorded in soleus but not flexor carpi radialis. Muscle Nerve 28, 609–614. doi: 10.1002/mus.10479, PMID: 14571464

[ref46] Mumford S. L. Steiner A. Z. Pollack A. Z. Perkins N. J. Filiberto A. C. Albert P. S.. (2012). The utility of menstrual cycle length as an indicator of cumulative hormonal exposure. J. Clin. Endocrinol. Metab. 97, E1871–E1879. doi: 10.1210/jc.2012-1350, PMID: 22837188 PMC3674299

[ref47] Osseward P. J. Pfaff S. L. (2019). Cell type and circuit modules in the spinal cord. Curr. Opin. Neurobiol. 56, 175–184. doi: 10.1016/j.conb.2019.03.003, PMID: 30954861 PMC8559966

[ref48] Papka R. E. Mowa C. N. (2003). Estrogen receptors in the spinal cord, sensory ganglia, and pelvic autonomic ganglia. Int. Rev. Cytol. 231, 91–127. doi: 10.1016/S0074-7696(03)31003-4 14713004

[ref49] Parducz A. Hajszan T. MacLusky N. J. Hoyk Z. Csakvari E. Kurunczi A.. (2006). Synaptic remodeling induced by gonadal hormones: neuronal plasticity as a mediator of neuroendocrine and behavioral responses to steroids. Neuroscience 138, 977–985. doi: 10.1016/j.neuroscience.2005.07.008, PMID: 16310961

[ref50] Reed B. G. Carr B. R. (2000). *The Normal menstrual cycle and the control of ovulation*. Available at: http://www.ncbi.nlm.nih.gov/pubmed/25905282. 25905282

[ref51] Sandrini G. Alfonsi E. Bono G. Facchinetti F. Montalbetti L. Nappi G. (1986a). Circadian variations of human flexion reflex. Pain 25, 403–410. doi: 10.1016/0304-3959(86)90245-9, PMID: 2944057

[ref52] Sandrini G. Alfonsi E. De Rysky C. Marini S. Facchinetti F. Nappi G. (1986b). Evidence for serotonin-S2 receptor involvement in analgesia in humans. Eur. J. Pharmacol. 130, 311–314. doi: 10.1016/0014-2999(86)90283-9, PMID: 3098573

[ref53] Sandrini G. Serrao M. Rossi P. Romaniello A. Cruccu G. Willer J. C. (2005). The lower limb flexion reflex in humans. Prog. Neurobiol. 77, 353–395. doi: 10.1016/j.pneurobio.2005.11.003 16386347

[ref54] Schock H. Zeleniuch-Jacquotte A. Lundin E. Grankvist K. Lakso H. Å. Idahl A.. (2016). Hormone concentrations throughout uncomplicated pregnancies: a longitudinal study. BMC Pregnancy Childbirth 16:146. doi: 10.1186/s12884-016-0937-5, PMID: 27377060 PMC4932669

[ref55] Sehested A. (2000). Serum inhibin a and inhibin B in healthy Prepubertal, pubertal, and adolescent girls and adult women: relation to age, stage of puberty, menstrual cycle, follicle-stimulating hormone, luteinizing hormone, and estradiol levels. J. Clin. Endocrinol. Metab. 85, 1634–1640. doi: 10.1210/jc.85.4.1634, PMID: 10770209

[ref56] Sellers K. Raval P. Srivastava D. P. (2015). Molecular signature of rapid estrogen regulation of synaptic connectivity and cognition. Front. Neuroendocrinol. 36, 72–89. doi: 10.1016/j.yfrne.2014.08.001, PMID: 25159586

[ref57] Shahani B. (1970). Flexor reflex afferent nerve fibres in man. J. Neurol. Neurosurg. Psychiatry 33, 786–791. doi: 10.1136/jnnp.33.6.786, PMID: 5531898 PMC493593

[ref58] Shughrue P. J. Lane M. V. Merchenthaler I. (1997). Comparative distribution of estrogen receptor-α and -β mRNA in the rat central nervous system. J. Comp. Neurol. 388, 507–525. doi: 10.1002/(SICI)1096-9861(19971201)388:4<507::AID-CNE1>3.0.CO;2-6, PMID: 9388012

[ref59] Shvetcov A. Ruitenberg M. J. Delerue F. Gold W. A. Brown D. A. Finney C. A. (2023). The neuroprotective effects of estrogen and estrogenic compounds in spinal cord injury. Neurosci. Biobehav. Rev. 146:105074. doi: 10.1016/j.neubiorev.2023.105074, PMID: 36736846

[ref60] Smith M. J. Adams L. F. Schmidt P. J. Rubinow D. R. Wassermann E. M. (2002). Effects of ovarian hormones on human cortical excitability. Ann. Neurol. 51, 599–603. doi: 10.1002/ana.10180, PMID: 12112106

[ref61] Smith S. S. Woolley C. S. (2004). Cellular and molecular effects of steroid hormones on CNS excitability. Cleve. Clin. J. Med. 71, S4–S10. doi: 10.3949/ccjm.71.suppl_2.s4 15379294

[ref62] Sousa A. S. P. Valente I. Pinto A. Santos R. (2019). Reliability of two methods for identifying the timing of medium latency responses in subjects with and without chronic ankle instability. Sci. Rep. 9:3115. doi: 10.1038/s41598-019-40073-z 30816323 PMC6395691

[ref63] Sribnick E. A. Wingrave J. M. Matzelle D. D. Ray S. K. Banik N. L. (2003). Estrogen as a neuroprotective agent in the treatment of spinal cord injury. Ann New York Acad Sci 993, 125–133. doi: 10.1111/j.1749-6632.2003.tb07521.x 12853305

[ref64] Srivastava D. P. (2012). Two-step wiring plasticity - a mechanism for estrogen-induced rewiring of cortical circuits. J. Steroid Biochem. Mol. Biol. 131, 17–23. doi: 10.1016/j.jsbmb.2012.01.006, PMID: 22349412

[ref65] Takanami K. Sakamoto H. Matsuda K. I. Hosokawa K. Nishi M. Prossnitz E. R.. (2010). Expression of G protein-coupled receptor 30 in the spinal somatosensory system. Brain Res. 1310, 17–28. doi: 10.1016/j.brainres.2009.11.004, PMID: 19912997 PMC6660911

[ref66] Tassorelli C. Sandrini G. Proietti Cecchini A. Nappi R. E. Sances G. Martignoni E. (2002). Changes in nociceptive flexion reflex threshold across the menstrual cycle in healthy women. Psychosom. Med. 64, 621–626. doi: 10.1097/01.PSY.0000021945.35402.0D 12140352

[ref67] Thomas A. J. Nockels R. P. Pan H. Q. Shaffrey C. I. Chopp M. (1999). Progesterone is neuroprotective after acute experimental spinal cord trauma in rats. Spine (Phila Pa 1976) 24, 2134–2138. doi: 10.1097/00007632-199910150-00013, PMID: 10543012

[ref68] Twyman R. E. Macdonald R. L. (1992). Neurosteroid regulation of GABAA receptor single-channel kinetic properties of mouse spinal cord neurons in culture. J. Physiol. 456, 215–245. doi: 10.1113/jphysiol.1992.sp019334, PMID: 1338096 PMC1175679

[ref69] Van Wezel B. M. H. Ottenhoff F. A. M. Duysens J. (1997). Dynamic control of location-specific information in tactile cutaneous reflexes from the foot during human walking. J. Neurosci. 17, 3804–3814. doi: 10.1523/jneurosci.17-10-03804.1997, PMID: 9133399 PMC6573668

[ref70] van Wezel B. M. H. van Engelen B. G. M. Gabreëls F. J. M. Gabreëls-Festen A. A. W. M. Duysens J. (2000). Aβ fibers mediate cutaneous reflexes during human walking. J. Neurophysiol. 83, 2980–2986. doi: 10.1152/jn.2000.83.5.2980 10805693

[ref71] VanderHorst V. G. J. M. Gustafsson J. Å. Ulfhake B. (2005). Estrogen receptor-α and -β immunoreactive neurons in the brainstem and spinal cord of male and female mice: relationships to monoaminergic, cholinergic, and spinal projection systems. J. Comp. Neurol. 488, 152–179. doi: 10.1002/cne.20569, PMID: 15924341

[ref72] Veale J. L. Mark R. F. Rees S. (1973). Differential sensitivity of motor and sensory fibres in human ulnar nerve. J. Neurol. Neurosurg. Psychiatry 36, 75–86. doi: 10.1136/jnnp.36.1.75, PMID: 4348037 PMC494280

[ref73] von Dincklage F. Benzke M. Rehberg B. Baars J. H. (2007). Ethanol reduces motoneuronal excitability and increases presynaptic inhibition of Ia afferents in the human spinal cord. Brain Res. 1173, 78–83. doi: 10.1016/j.brainres.2007.07.052, PMID: 17825271

[ref74] Walton C. Kalmar J. Cafarelli E. (2003). Caffeine increases spinal excitability in humans. Muscle Nerve 28, 359–364. doi: 10.1002/mus.10457, PMID: 12929197

[ref75] Warren S. G. Humphreys A. G. Juraska J. M. Greenough W. T. (1995). LTP varies across the estrous cycle: enhanced synaptic plasticity in proestrus rats. Brain Res. 703, 26–30. doi: 10.1016/0006-8993(95)01059-9, PMID: 8719612

[ref76] Weiland N. G. Orchinik M. (1995). Specific subunit mRNAs of the GABAA receptor are regulated by progesterone in subfields of the hippocampus. Mol. Brain Res. 32, 271–278. doi: 10.1016/0169-328X(95)00087-9, PMID: 7500838

[ref77] Whalen R. E. (1974). Estrogen-progesterone induction of mating in female rats. Horm. Behav. 5, 157–162. doi: 10.1016/0018-506X(74)90040-3, PMID: 4847184

[ref78] Windhorst U. (2021). Spinal cord circuits: models and reality. Neurophysiology 53, 142–222. doi: 10.1007/s11062-022-09927-7

[ref79] Woolley C. S. McEwen B. S. (1993). Roles of estradiol and progesterone in regulation of hippocampal dendritic spine density during the estrous cycle in the rat. J. Comp. Neurol. 336, 293–306. doi: 10.1002/cne.903360210, PMID: 8245220

[ref80] Wright D. W. Kellermann A. L. Hertzberg V. S. Clark P. L. Frankel M. Goldstein F. C.. (2007). ProTECT: a randomized clinical trial of progesterone for acute traumatic brain injury. Ann. Emerg. Med. 49, 391–402.e2. doi: 10.1016/j.annemergmed.2006.07.932, PMID: 17011666

